# The Role of GSK-3β in the Regulation of Protein Turnover, Myosin Phenotype, and Oxidative Capacity in Skeletal Muscle under Disuse Conditions

**DOI:** 10.3390/ijms22105081

**Published:** 2021-05-11

**Authors:** Timur M. Mirzoev, Kristina A. Sharlo, Boris S. Shenkman

**Affiliations:** Myology Laboratory, Institute of Biomedical Problems RAS, 123007 Moscow, Russia; sharlokris@gmail.com (K.A.S.); bshenkman@mail.ru (B.S.S.)

**Keywords:** skeletal muscle, disuse, unloading, muscle recovery, GSK-3beta, protein synthesis, protein breakdown, myosin phenotype, oxidative capacity

## Abstract

Skeletal muscles, being one of the most abundant tissues in the body, are involved in many vital processes, such as locomotion, posture maintenance, respiration, glucose homeostasis, etc. Hence, the maintenance of skeletal muscle mass is crucial for overall health, prevention of various diseases, and contributes to an individual’s quality of life. Prolonged muscle inactivity/disuse (due to limb immobilization, mechanical ventilation, bedrest, spaceflight) represents one of the typical causes, leading to the loss of muscle mass and function. This disuse-induced muscle loss primarily results from repressed protein synthesis and increased proteolysis. Further, prolonged disuse results in slow-to-fast fiber-type transition, mitochondrial dysfunction and reduced oxidative capacity. Glycogen synthase kinase 3β (GSK-3β) is a key enzyme standing at the crossroads of various signaling pathways regulating a wide range of cellular processes. This review discusses various important roles of GSK-3β in the regulation of protein turnover, myosin phenotype, and oxidative capacity in skeletal muscles under disuse/unloading conditions and subsequent recovery. According to its vital functions, GSK-3β may represent a perspective therapeutic target in the treatment of muscle wasting induced by chronic disuse, aging, and a number of diseases.

## 1. Introduction

It is well-established that mechanical unloading/disuse results in a slow-to-fast fiber type transition and a significant reduction in the rate of muscle protein synthesis, increased proteolysis, and subsequent fiber atrophy (loss of muscle mass) [1,2,3]. Disuse-induced loss of skeletal muscle mass and function represents a serious issue for both clinical and space medicine. In order to combat unloading/disuse muscle wasting it is vital to understand intracellular molecular events which result in the development of skeletal muscle atrophy. Muscle atrophy is mainly linked to a depressed anabolism (protein synthesis) and enhanced catabolism (protein breakdown). Glycogen synthase kinase 3 (GSK3) is a key enzyme standing at the crossroads of various signaling pathways regulating a wide range of cellular processes in different tissues including skeletal muscle. Being a multifaceted Ser/Thr protein kinase known to phosphorylate several hundred substrates [4], GSK3 exists in 2 isoforms, GSK-3α and GSK-3β, among which GSK-3β is considered to be the most expressed and active isoform within skeletal muscles [5]. Given that GSK-3β is involved in the regulation of the key metabolic pathways in skeletal muscle, this enzyme may represent a perspective therapeutic target in the treatment of muscle wasting induced by chronic disuse and a number of diseases (cancer, rheumatoid arthritis, heart, and renal failure, chronic obstructive pulmonary disease, etc.) as well as aging (sarcopenia). In this review paper, we will focus on the effects of mechanical unloading and subsequent recovery on GSK-3β activity in a mammalian skeletal muscle as well as discuss recent advances concerning a potential role of this kinase in the regulation of protein synthesis and degradation, myosin phenotype, and oxidative capacity under disuse conditions. The present review takes a special place among other recently published papers on signaling mechanisms of disuse-induced muscle atrophy in a sense that our review is focused exclusively on the key role of GSK-3β in the regulation of protein turnover and myosin phenotype/oxidative capacity under various disuse conditions (hindlimb suspension, limb immobilization, dry immersion) in skeletal muscles of different types (soleus, gastrocnemius, tibialis anterior, vastus lateralis), and in different systems (cell culture, mouse, rat, human). Moreover, to our knowledge, there is a lack of reviews in the field dedicated to recent findings related to the role of GSK-3β in skeletal muscle metabolism during recovery from disuse atrophy.

## 2. Regulation of GSK-3β Activity

A detailed overview of the function and regulation of GSK-3β activity has been earlier provided in several excellent review articles [6,7] and, therefore, in the present paper only key mechanisms of GSK-3β regulation will be discussed in brief. The best-studied mechanism of GSK-3β regulation is its negative Ser9 phosphorylation, positive Tyr 216 phosphorylation as well as dephosphorylation of Ser residues. One of the best-known pathways involved in the negative GSK-3β phosphorylation is an IGF-1/insulin-dependent cascade, in which activated protein kinase B (AKT) is directly responsible for GSK-3β Ser9 phosphorylation and inhibition [8,9]. In addition, GSK-3β inactivation via Ser9 phosphorylation has been shown to be mediated by such kinases as cAMP-dependent protein kinase A (PKA) [10], gamma isoform of protein kinase C (PKC) [11], protein kinase D1 [12], and protein kinase G (PKG) [13]. Regulation of GSK-3β by PKG is known to be carried out via the classic NO/soluble guanylate cyclase (GC)/cyclic guanosine monophosphate (cGMP)/PKG signaling pathway [14,15]. It has been shown in vitro that GSK-3β can also be phosphorylated and inactivated by ribosomal protein S6 kinase (p70S6K) or mitogen-activated protein kinase (MAPK)-activated protein kinase-1 [16,17]. Furthermore, integrin-linked kinase (ILK) has been shown to directly phosphorylate GSK-3β (at a residue distinct from Ser9) and inhibit its activity, whereas the overexpression of kinase-deficient ILK is able to increase GSK-3 activity [18]. In contrast to inhibitory Ser9 phosphorylation, Tyr216 phosphorylation is able to render a stimulatory effect on GSK-3β activity by facilitating substrate accessibility and subsequent substrate phosphorylation [19,20]. However, there is evidence that pharmacological inhibition of GSK-3β leads to increased inhibitory Ser9 phosphorylation without affecting Tyr216 phosphorylation suggesting that the effect of negative Ser9 phosphorylation is able to override the effect of positive Tyr216 phosphorylation [21]. Notably, GSK-3β can be activated via dephosphorylation of the N-terminal Ser residues by protein phosphatases 1 and 2A [22,23].

It has been recently demonstrated that, apart from phosphorylation, GSK-3β activity can be modulated by S-nitrosylation [4]. Indeed, Wang et al. (2018) have shown a novel mechanism regulating GSK-3β function via S-nitrosylation at multiple Cys residues leading to the inhibition of cytosolic GSK-3β activity [4]. Importantly, S-nitrosylation of GSK-3β is independent of both its Ser9 phosphorylation state and NO/GC/cGMP/PKG pathway [4]. Moreover, S-nitrosylation leads to GSK-3β nuclear translocation, and nuclear S-nitrosylated GSK-3β can increase phosphorylation of numerous nuclear targets, despite inhibition of cytosolic substrate phosphorylation [4]. The latter finding confirms the notion that subcellular localization of GSK-3β can modulate its enzymatic activity.

In addition to posttranslational regulation via phosphorylation and S-nitrosylation, GSK-3β activity may depend upon protein-protein interactions. A typical example is a so-called “destruction complex” in the Wnt signaling pathway. This protein complex consists of axin, adenomatous polyposis coli (APC), casein kinase 1, and GSK-3β [6]. Following priming phosphorylation by casein kinase 1, GSK-3β can phosphorylate β-catenin, thereby marking it for degradation by proteasome [6]. It is important to note that the activity of GSK-3 bound to axin is not regulated by the GSK-3 Ser phosphorylation [6].

**Figure 1:**
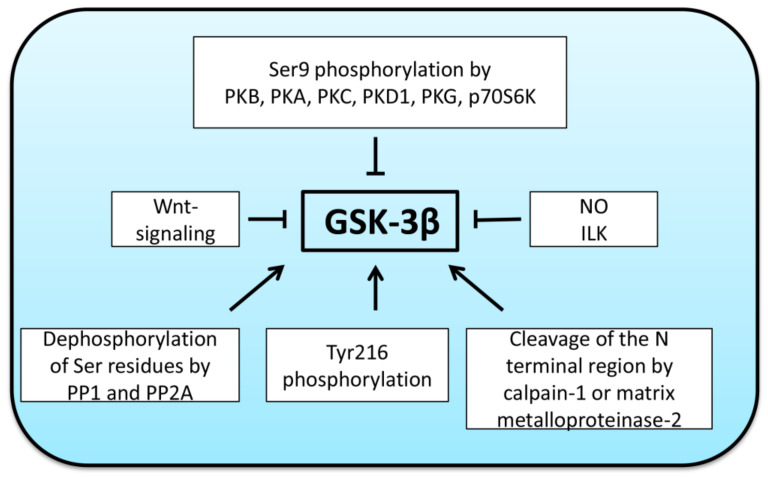
Positive and negative regulators of GSK-3β activity. Notably, apart from regulators showed in the figure, GSK-3β activity can also depend upon subcellular localization and protein-protein interactions. PKA—protein kinase A, PKA—protein kinase B (AKT), PKC—protein kinase C, PKD1—protein kinase D1, PKG—protein kinase G, p70S6K—ribosomal protein S6 kinase, NO—nitric oxide, ILK—integrin-linked kinase, PP1 and PP2A—protein phosphatases 1 and 2A.

Another mechanism of GSK-3β regulation includes proteolytic cleavage of the N-terminal region (which contains the regulatory Ser9 residue) by calpain-1 [24] or matrix metalloproteinase-2 [25], resulting in the activation of GSK-3β kinase activity. A schematic overview of the key positive and negative regulators of GSK-3β activity is presented in Figure 1.

Thus, the regulation of GSK-3β activity is a complex process, which can be mediated by different mechanisms including phosphorylation/dephosphorylation, S-nitrosylation, subcellular localization, protein–protein interactions, as well as proteolytic cleavage.

## 3. The Role of GSK-3β in the Regulation of Protein Synthesis and Breakdown

The maintenance of skeletal muscle mass depends on the balance between anabolic processes associated with protein synthesis and catabolic processes related to protein breakdown. Protein synthesis is determined by both translational efficiency (the rate of protein synthesis per unit RNA), and translational capacity (the total ribosomal content per unit tissue) [26]. Muscle protein degradation is mainly achieved via such pathways as calpain-dependent, ubiquitin-proteasome pathway (UPP) and autophagy–lysosomal pathway [27,28]. Literature suggests that GSK-3β may be involved in the regulation of both anabolic and proteolytic signaling pathways being an endogenous inhibitor of protein synthesis and promoter of protein degradation.

At the level of translational efficiency, GSK-3β can negatively regulate mRNA translation initiation via phosphorylation and inhibition of the epsilon subunit of the eukaryotic initiation factor 2B (eIF2B-epsilon). This initiation factor is needed for GDP for GTP exchange on eukaryotic initiation factor 2 (eIF2), thereby allowing Met-tRNAi binding to the start codon (AUG) in order to initiate the process of mRNA translation [29]. It has been shown that inactivation of GSK-3β leads to dephosphorylation of eIF2B-epsilon at Ser 539/540, which, in turn, promotes the initiation of protein synthesis [30,31]. In the absence of anabolic factors/hormones, GSK-3β has also been shown to inhibit mechanistic target of rapamycin complex-1 (mTORC1), a key regulator of protein synthesis and cell growth, via phosphorylation of tuberous sclerosis complex-2 (TSC2), an endogenous mTORC1 inhibitor. Inoki et al. (2006) have demonstrated that GSK-3 can inhibit mTOR-signaling via phosphorylating TSC2 in a manner dependent on AMP-activated protein kinase (AMPK)-priming phosphorylation [32]. Interestingly, under anabolic conditions, GSK-3 was shown to interact with the AMPK β regulatory subunit (AMPK is known as a negative regulator of protein synthesis) and phosphorylate the AMPK α catalytic subunit, thereby inhibiting AMPK kinase activity [33]. The authors of the latter finding suggest that GSK-3 in the AMPK heterotrimeric complex may play an important role in sensing insulin-like growth factor-1 (IGF-1)/insulin signaling to modulate AMPK activity during transitions between catabolic and anabolic states [33]. Activated GSK-3 can also contribute to the suppression of the anabolic IGF-1/IRS-1/PI3K/AKT signaling pathway via negative Ser phosphorylation of both insulin-like growth factor 1 receptor (IGF-1R) [34] and insulin receptor substrate 1 (IRS-1) [35,36].

Apart from translational efficiency, GSK-3β can be involved in the control of translational capacity via regulation of β-catenin and phosphorylation of transcription factor myelocytomatosis oncogene (c-Myc). This transcription factor (c-Myc) can promote transcription of the 47S pre-rRNA via activation of selective factor 1 and upstream binding factor that bind to the rDNA promoter and stabilize the initiation complex. Moreover, c-Myc participates in the activation of genes encoding ribosomal proteins of ribosomal subunits and promotes transcriptional activity of RNA polymerase III [37]. Dephosphorylated β-catenin is able to translocate to the nucleus and activate growth-control genes (including c-Myc) during overload-induced hypertrophy in skeletal muscles [38]. As mentioned above, GSK-3β can negatively phosphorylate β-catenin preventing it from nuclear translocation and thereby targeting β-catenin for proteasomal degradation. Furthermore, it has been shown that GSK-3β can directly phosphorylate c-Myc on Thr58, resulting in c-Myc ubiquitination (possibly, by muscle atrophy F-box (MAFbx), a well-known E3 ubiquitin ligase [39]) and subsequent degradation by the proteasome [40,41]. Thus, GSK-3β can be involved in the regulation of signaling pathways responsible for both translational efficiency and translational capacity. The role of GSK-3β in the suppression of the rates of protein synthesis was shown in an isolated skeletal muscle [42] as well as in rat cardiomyocytes [43]. In addition, it was demonstrated that the expression of dominant-negative form of GSK-3β results in a hypertrophy of cultured myotubes [44].

With regard to proteolysis, it was shown that treatment of isolated rat skeletal muscle (extensor digitorum longus) from septic rats with GSK-3β inhibitors (LiCl or thiadiazolidinone-8, TDZD-8) is able to significantly reduce the rate of protein breakdown [45]. In addition, the use of GSK-3β inhibitors prevented an increase in the mRNA expression of MAFbx and MuRF1 (muscle-specific E3 ubiquitin ligases) in dexamethasone-treated myotubes [45]. These data suggest that inhibition of GSK-3β may prevent protein degradation via suppression of UPP-mediated proteolysis. These data are in line with the results obtained by Li et al. (2005) who also demonstrated that IGF-I-induced inhibition of dexamethasone-induced proteolysis in cultured L6 myotubes is dependent on AKT/GSK-3β signaling pathway [46]. Moreover, Verhees et al. (2011) clearly demonstrated that GSK-3β-deficient C2C12 myotubes had repressed expression levels of both MAFbx and MuRF1 [47]. In addition, application of both LiCl and TDZD-8 was able to reduce burn-induced protein breakdown in isolated rat extensor digitorum longus muscle [48]. Bertsch et al. (2011) showed that incubation of septic epitrochlearis muscle with lithium reversed an increased GSK-3β activity and decreased proteolysis to basal non-septic values [42]. Application of lithium also partially reversed an elevated 26S proteasome activity in septic muscle, but did not affect the sepsis-induced increase in the expression of muscle-specific E3 ubiquitin ligases [42]. As for regulation of autophagy, incubation of skeletal muscle with lithium did not alter a sepsis-induced increase in the LC3-II/LC3-I ratio [42].

**Figure 2:**
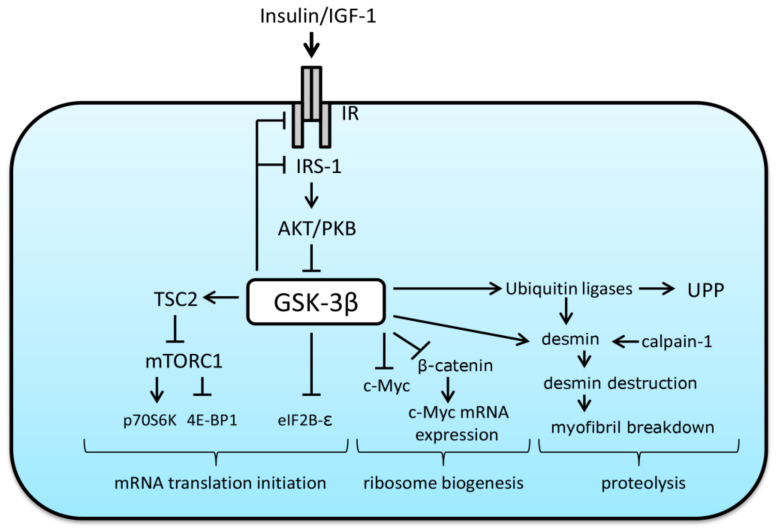
Involvement of GSK-3β in the regulation of anabolic and catabolic signaling pathways. Please see description in the text above. Arrows indicate stimulatory signals, whereas T bars represent inhibitory signals. IGF-1—insulin-like growth factor-1, IR—insulin/insulin-like growth factor receptor, IRS-1—insulin receptor substrate-1, PKB—protein kinase B, UPP—ubiquitin-proteasome pathway, TSC2—tuberous sclerosis complex 2, mTORC1—mammalian/mechanistic target of rapamycin complex 1, p70S6K—ribosomal protein S6 kinase, 4E-BP1—eukaryotic initiation factor 4E binding protein, eIF2B-ε—epsilon subunit of the eukaryotic initiation factor 2B, c-Myc—c-myelocytomatosis oncogene (transcription factor).

Skeletal muscle atrophy due to prolonged disuse, aging, or certain disease states largely results from a significant degradation of myofibrillar proteins and subsequent loss of myofibrils. Aweida et al. (2018) have recently revealed a mechanism promoting disassembly of desmin filaments, resulting in myofibril destruction during denervation- or fasting-induced muscle atrophy [49]. The induction of this mechanism is associated with the activity of GSK-3β and calpain-1. The authors suggested that phosphorylation of desmin by GSK-3β precedes and promotes ubiquitination by Trim32 and subsequent depolymerization by calpain-1, leading to myofibril disassembly and fiber atrophy [49]. Thus, GSK-3β may play a crucial role in the initiation of destruction of myofibrillar proteins under atrophic conditions. Possible roles of GSK-3β in the regulation of both anabolic and catabolic processes are depicted in Figure 2.

Based on the data presented above, we can speculate that, under natural conditions, a certain degree of GSK-3β Ser9 phosphorylation may be necessary for maintaining a stable functioning/balance of signaling networks involved in the regulation of protein synthesis and proteolysis. However, such conditions as mechanical unloading/disuse would significantly influence GSK-3β phosphorylation status leading to an imbalance between anabolic and catabolic processes in skeletal muscle. The effect of mechanical unloading on GSK-3β Ser9 phosphorylation in mammalian skeletal muscles is discussed below.

## 4. Impact of Mechanical Unloading on GSK-3β Activity and Possible Roles of this Enzyme in the Regulation of Protein Turnover in Mammalian Skeletal Muscles under Disuse Conditions and Subsequent Recovery

It has been shown that even short-term hindlimb unloading (HU) (1–7 days) results in a significant decrease in GSK-3β Ser9 phosphorylation in rat soleus muscle total protein and nuclear protein fractions [50,51,52,53]. Two-week HU has been shown to either reduce GSK-3β Ser9 phosphorylation in rat soleus [52,54,55] or not affect GSK-3β phosphorylation status in mouse soleus [56] or rat soleus muscle [57,58]. Childs et al. (2003) did not observe any changes in GSK-3β (Ser9) phosphorylation in rat soleus muscle following 10-day hindlimb immobilization [59]. It has also been shown that chronic exposure to HU (28 or 38 days) is able to induce a significant decrease in the phospho-GSK-3β/total GSK-3β ratio in soleus muscles of rats [52,60]. Thus, in most cases mechanical unloading induces a reduction in GSK-3β Ser9 phosphorylation in rat postural soleus muscle indicating a higher activity of this enzyme. In that regard, a question that arises is what upstream kinases might be responsible for the decreased Ser9 phosphorylation under unloading conditions? It was earlier demonstrated that such kinases as PKB (AKT) [51,61,62] and PKC [63] are significantly downregulated in rat soleus muscle following both earlier (3 days) and later (14 days) stages of HU. At the same time, it was recently demonstrated that GSK-3β Ser9 phosphorylation during HU can be modulated independently of AKT Ser473 phosphorylation [61]. Preliminary evidences suggest that PKD1 and PKA do not contribute to a HU-induced decrease in GSK-3β Ser9 phosphorylation, at least within the first 24h of unloading, since the activity of these kinases is upregulated in rat soleus during an acute stage of HU [64] (Vilchinskaya et al., unpublished work). It is also possible that a reduction in GSK-3β Ser9 phosphorylation during disuse conditions could be associated with NO-dependent (cGMP)/PKG signaling pathway, since HU promotes a significant decrease in NO content in rat soleus muscle [65,66].

A response of GSK-3β phosphorylation and other anabolic and catabolic markers to plantar mechanical stimulation (PMS) (4 h/day) in rat soleus muscle during the initial period of HU (1 and 3 days) has been recently investigated in our laboratory [61]. This type of mechanical stimulation (PMS) can induce an increase in the neuromuscular activity in postural muscles (such as the soleus) via mechanisms of support afferentation [67]. The application of PMS during 3-day HU was able to fully prevent a reduction in GSK-3β Ser9 phosphorylation and MuRF-1 upregulation and partially attenuate a decrease in the rate of protein synthesis in rat soleus muscle [61]. These data suggest that PMS-induced tonic electrical activity of the unloaded soleus muscle is able to suppress HU-induced activity of GSK-3β, an endogenous inhibitor of anabolic processes. Furthermore, at later stages of mechanical unloading (7 days) this PMS-induced effect on GSK-3β activity and protein synthesis may be mediated by NO-synthase (NOS) activity (Tyganov et al., 2021, under review).

Pansters et al. (2015) demonstrated that muscle-specific deletion of GSK-3β did not significantly affect markers of protein turnover in mouse gastrocnemius muscle following 14-day HU [68]. In particular, mTOR (Ser2448) phosphorylation as well as phosphorylation of eukaryotic translation initiation factor 4E-binding protein 1 (4E-BP1) and p70S6K were unchanged after 14 days of unloading in gastrocnemius muscle with GSK-3β deletion [68]. At baseline, MAFbx mRNA expression was lower in GSK-3β knock-out (GSK-3β KO) mice than in wild-type (WT) animals, and increased similarly after 14-day unloading in both WT and GSK-3β KO mice [68]. MuRF1 mRNA expression did not differ between genotypes at baseline, and did not alter after 14-day hindlimb suspension in either WT or GSK-3β KO animals [68].

**Figure 3:**
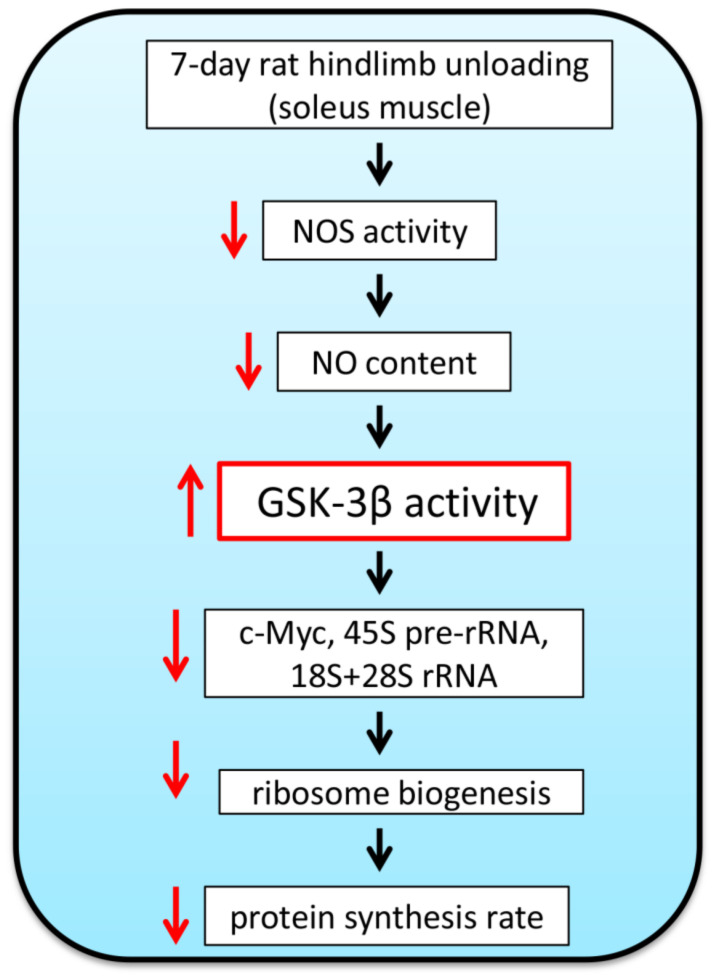
Simplified diagram showing the role of NO-dependent regulation of ribosome biogenesis via modulation of GSK-3β activity in rat soleus muscle under disuse conditions. One-week hindlimb unloading results in a significant reduction in NO content in rat soleus muscle. This event leads to a significant increase in GSK-3β activity (a decrease in GSK-3β Ser9 phosphorylation) which, in turn, can negatively affect ribosome biogenesis and the rate of muscle protein synthesis. Upwards red arrow indicates upregulation; downwards red arrows indicate downregulation. NOS—nitric oxide synthase, NO—nitric oxide, c-Myc—c-myelocytomatosis oncogene (transcription factor), rRNA—ribosomal RNA.

Recent data from our laboratory suggest that GSK-3β activity can be involved in the regulation of translational capacity in rat soleus muscle during 7-day HU. Treatment of rats with GSK-3beta inhibitor (AR-A014418, daily injections during 7-day HU, 4mg/kg) prevented HU-induced increase in GS1 (Ser641) phosphorylation, which was indicative of GSK-3β inhibition. Administration of GSK-3beta inhibitor also prevented an unloading-induced downregulation of c-Myc mRNA expression as well as decreases in the levels of 45S pre-rRNA and 18S + 28S rRNAs (unpublished data). These AR-A014418-induced alterations in the markers of ribosome biogenesis were paralleled with partial prevention of a decrease in the rate of protein synthesis (unpublished data). A diagram depicting an NO-dependent role of GSK-3β in the regulation of ribosome biogenesis in rat soleus muscle during hindlimb unloading is presented in Figure 3.

In humans, leg immobilization for 48 h resulted in a significant 21% decline in GSK-3β (Ser9) phosphorylation in vastus lateralis muscle [69]. However, 14 days of unilateral knee immobilization did not affect total and phospho-GSK-3β protein content in vastus lateralis of young healthy subjects [70]. Preliminary data from our laboratory have recently shown that 21-day mechanical unloading via dry immersion leads to an increase in GSK-3β activity in human soleus muscle as assessed by phosphorylation of both GSK-3β and its direct substrate glycogen synthase 1 (unpublished data).

To our knowledge, Childs et al. (2003) were the first who measured the content of phospho- and total GSK-3β in rat skeletal muscle during recovery from disuse-induced atrophy [59]. That study revealed a significant increase in phospho-GSK-3β (Ser9) in rat soleus muscle on the 6th and 15th recovery days following 10-day hindlimb cast immobilization [59]. In murine soleus muscle, 3 and 5 days of reloading after 14-day HU resulted in a significant increase in phospho-GSK-3β (Ser9)-to-total GSK-3β ratio compared to the baseline levels [56]. Increased GSK-3β phosphorylation during reloading was corroborated by significantly decreased GSK-3β kinase activity (67 ± 2% of baseline) following 5-day reloading [56]. Interestingly, in contrast to soleus muscle, GSK-3β phosphorylation and kinase activity in plantaris muscle were not affected during the period of reloading compared with the control values [56]. The latter observation suggests that GSK-3β response to reloading period following unloading conditions can be muscle-specific. It has been also suggested that regenerative response (myoblast proliferation, differentiation and increased expression of muscle regulatory factors) of the mouse soleus muscle during reloading after HU may be negatively regulated by GSK-3β [56]. In line with the above studies, our laboratory has also observed a significant increase in GSK-3β (Ser9) phosphorylation in rat soleus muscle on the third day of recovery from disuse atrophy [57,58]. Baehr et al. (2016) also revealed that in soleus muscle of adult 9 month old rats GSK-3β (Ser9) phosphorylation is significantly elevated on day 3 of reloading after HU [54]. Of note, in tibialis anterior muscle of adult rats Ser9 phosphorylation of GSK-3β remained increased until 14-day reloading [54].

In an interesting study by Pansters et al. (2015) wild-type mice (WT) and mice with muscle-specific GSK-3β ablation (MGSK-3β KO) were subjected to HU and subsequent 1, 2, 3, or 5 days of reloading [68]. The authors have shown that the recovery of soleus muscle mass and fiber cross-sectional area (CSA) after 5 days of reloading in MGSK-3β KO mice were more pronounced as compared to WT. In gastrocnemius muscle, muscle specific deletion of GSK-3β also enhanced myogenesis-associated gene expression (MyoD and myogenin) upon reloading. Of note, GSK-3β deficiency was not able to prevent HU-induced soleus muscle atrophy [68]. Moreover, a reduction in CSA after 14-day HU was more evident in soleus of MGSK-3β KO mice relative to WT mice [68]. Interestingly, the same research group has recently demonstrated that GSK-3 inactivation via Ser9 or Ser21 phosphorylation is not required for reloading-induced muscle mass recovery [71]. This finding was obtained in soleus muscles of whole-body constitutively active Ser21/9 GSK-3α/β knock-in mice [71]. Thus, although GSK-3β activity can suppress soleus mass recovery after a period of inactivity (hindlimb suspension), suppressive actions of GSK-3β do not appear to be regulated by Ser9 phosphorylation.

The above described changes in GSK-3β (Ser9) phosphorylation due to mechanical unloading and subsequent reloading (recovery) in mammalian skeletal muscles are summarized in Table 1.

It is evident from the table above that GSK-3β activation in soleus muscle following 14 days of disuse/unloading might be species-specific. In mouse soleus muscle, in contrast to rat soleus muscle, no changes in GSK-3β phosphorylation were observed after 2-week HU. It is possible that this discrepancy could be associated with varying fiber-type composition of the soleus muscle in mice and rats (rat soleus muscle is known to be “slower” than that of mouse). The observed differences in GSK-3β response to 14-day unloading in rat soleus and tibialis anterior muscles could be due to varied physiological functions (ankle extensors vs. ankle flexors) as well as different metabolic profiles based upon fiber-type composition (slow soleus vs. fast tibialis anterior). Following longer periods of unloading (38 days), differences in GSK-3β phosphorylation were observed in skeletal muscles with differing myosin phenotype (rat soleus vs. rat gastrocnemius). During the recovery period (reloading) at various time points a different GSK-3β response in skeletal muscles that differ in fiber-type composition/myosin phenotype was also documented. Thus, literature analysis suggests that in order to gain a complete picture of the disuse-induced changes, measurements of posttranslational modifications/activities of the key endogenous metabolic regulators, such as GSK-3β, should be performed for different muscle types and at multiple time points during the course of both unloading and subsequent reloading.

## 5. Impact of GSK-3β Activity on Fiber-Type Transitions and Oxidative Capacity in Skeletal Muscles under Mechanical Unloading and Subsequent Reloading

Skeletal muscles are composed of fibers with different functional characteristics. Slow-type fibers have a higher fatigue resistance, a longer duration of contraction, but also a lower maximum contraction force. In humans, slow-twitch fibers have a higher mitochondrial density and increased mitochondrial enzyme content than fast-twitch fibers, as well as increased content of myoglobin, resulting in a higher rate of aerobic metabolism in this type of fibers [72]. Fast-type muscle fibers demonstrate a higher contraction force and content of glycolytic enzymes, but show an increased fatigability. There are several classifications of skeletal muscle fiber types, such as ATPase staining-based method [73], but nowadays the most accurate method of fiber-type classification is based on the analysis of the predominant isoform of myosin heavy chain (MyHC) within muscle fibers [74]. There are four major MyHC isoforms in mammalian skeletal muscles: the “slow” MyHC I(β) and the three “fast” IIa-, IIx-, and IIb- MyHCs [75]. The predominance of one of these isoforms in a skeletal muscle determines the phenotype of the muscle, and the myosin phenotype of a skeletal fiber determines muscle metabolism. MyHC I(β) and MyHC IIa-containing fibers are known as oxidative fibers, glycolytic fibers contain MyHC IIb isoform, and fibers expressing MyHC IId/x have intermediate number of glycolytic and mitochondrial enzymes [76,77]. Some fibers may express more than one MyHC isoform (which may serve as a marker of an ongoing fiber-type transition) and are considered to be as hybrid muscle fibers [78]. A growing body of evidence suggests that the expression of a distinct MyHC isoform, especially MyHC I(β) may lead to an upregulation of specific genes, orchestrating the formation of oxidative, slow-type muscle fiber phenotype [79,80,81,82,83].

Under conditions of mechanical unloading, hindlimb postural muscles (such as the soleus muscle) undergo a number of changes associated with the elimination of support afferentation followed by almost a complete reduction in muscle contractile activity. Transformation of the myosin phenotype in mammalian postural muscles is observed under both real and simulated microgravity [2,84,85,86,87,88,89,90]. As early as after the first hours of mechanical unloading a significant decrease in MyHC I mRNA expression can occur [91,92,93,94]. At the later stages of unloading, slow-to-fast myosin phenotype transformation of the soleus muscle (containing up to 85% of slow-type fibers in rats and humans) takes place [1,95]. These changes lead to an increased fatigue and a decrease in maximum contraction force in soleus muscle [86,96]. In soleus and adductor longus muscles, a 20–25% decrease in the amount of type I fibers was observed following 12.5–14 days of spaceflight [85,97]. In our laboratory, after 7-day exposure to dry-immersion conditions, a significant decrease in the proportion of fibers expressing “slow” MyHC isoform was observed in human soleus muscle [98]. The unloading-induced changes in myosin expression are accompanied by downregulation of mitochondrial density and the content of mitochondrial respiratory chain enzymes in both fast and slow skeletal muscles [99,100,101].

Expression of different MyHC isoforms critically depends on the pattern of electrical activity of skeletal muscle. Calcineurin/NFATc1 signaling pathway is one of the key mechanisms connecting MyHC I gene transcription to skeletal muscle activity. Calcineurin (CaN) is a serine/threonine phosphatase localized to the sarcomeric Z-disc [102]. When interacting with the calcium–calmodulin complex, CaN can dephosphorylate NFATc1-4 (nuclear factor of activated T-cells, cytoplasmic), and induce its nuclear translocation [103,104]. Inside the nuclei NFATc1 binds to MyHC I(β) promoter and induces its transcription [105]. NFAT transcriptional factors (at least, NFATc1 and NFATc2) are downstream targets of GSK-3β: GSK-3β phosphorylates multiple serine residues on NFAT molecules, leading to conformational changes, opening nuclear export signal (NES) sites of the NFAT molecule resulting in nuclear export of NFAT [106,107]. Thus, GSK-3β is able to counteract calcineurin activity.

GSK-3β activity is known to significantly contribute to the nuclear export of NFATc1 in murine muscle fibers following slow fiber type electrical stimulation [108]. Martins and colleagues showed that nitric oxide synthase (NOS) inhibition during muscle low-frequency electrical stimulation prevents fast-to-slow fiber-type transformation and an increase in MyHC I mRNA transcription via inhibition of GSK-3β-induced NFATc1 nuclear export [109]. The detailed mechanism of GSK-3β-dependent regulation of MyHC I gene transcription was revealed by Drenning and colleagues (2008). They showed that in C2C12 myotubes, inhibition of guanylate cyclase, as well as administration of NOS inhibitor, prevented a calcium-induced increase in NFAT-dependent transcription. At the same time, the introduction of a NO donor enhanced NFAT-dependent transcription and increased the content of NFAT in muscle nuclei as well as the level of GSK-3β phosphorylation, while the introduction of NOS inhibitor blocked all the effects induced by administration of NO donor [15]. These data suggest that GSK-3β activity is a powerful inhibitor of MyHC I gene transcription in skeletal muscle both in vitro and in vivo.

The role of GSK-3β activity under muscle mechanical unloading was studied using rat hindlimb suspension (HS) model. Plantar mechanical stimulation (PMS) during HS is able to restore support afferentation leading to the activation of slow motor units followed by increased postural muscle electrical activity [110,111,112]. In our laboratory it was shown that GSK-3β Ser9 phosphorylation decreases after the first 24 h of HS, which is accompanied by two-fold decrease in NFATc1 myonuclear content and 90% decrease in NFAT-dependent transcriptional activity (accessed by MCIP1.4 mRNA content). One 4-h session of PMS during the first 24-h of HS led to a prevention of a decrease in GSK-3β Ser9 phosphorylation as well as nuclear NFATc1 content in rat soleus muscle. It also partially prevented a HS-induced decrease in slow MyHC mRNA expression. Daily PMS sessions during 3-day HS had the same effect on GSK-3β Ser9 phosphorylation and MyHC mRNA expression, and also partially restored NFAT-dependent transcription activity, and content of neuronal NO-synthase (nNOS), and PGC-1-alpha in total protein fraction in rat soleus muscle [50,61]. Seven days of PMS during HS also prevented a reduction in GSK-3β Ser9 phosphorylation, NFATc1 nuclear content as well as MyHC I and MCIP1.4 mRNA expression. Importantly, all these effects were accompanied by an elevation of cytoplasmic NO content. Moreover, immunohistochemical analysis showed that application of PMS during 7-day unloading prevented slow-to-fast fiber-type transition in rat soleus. All these effects of PMS were completely abrogated in the group of animals in which PMS sessions were accompanied by administration of NOS inhibitor L-NAME [66]. Direct inhibition of GSK-3β activity during 7-day HS as well as NO donor L-arginine administration also led to NFATc1 nuclear accumulation and prevention of a decrease in MyHC I mRNA expression in rat soleus muscle [113]. These data suggest that GSK-3β inhibition is enough to prevent an unloading-induced downregulation of slow myosin mRNA expression. Thus, it was shown that NO may serve as a key regulator of activity-dependent GSK-3β inhibition, which is critical for NFAT/MyHCI signaling in rat soleus muscle under mechanical unloading. Both L-arginine administration and PMS during 7-day HS partially prevented downregulation of MyHC IIa mRNA transcription [66,114]. Previously it was shown that GSK-3β inhibition is able to upregulate the expression of slow myosin in chicken skeletal muscle [115] and MyHC IIa in goat satellite cells [116], however it is unclear why 3 days of PMS during unloading led to GSK-3β inhibition but did not activate MyHC IIa transcription in rat soleus muscle.

GSK-3β was also shown to be a potent regulator of mitochondrial biogenesis in skeletal muscle. In vitro, both knockdown as well as pharmacological inhibition of GSK-3β increased the expression levels of downstream targets of peroxisome proliferator-activated receptor gamma coactivator 1-alpha (PGC-1α) (a key controller of mitochondrial biogenesis), potentiated myogenic differentiation-associated increases in mitochondrial respiration, increased mitochondrial DNA copy number, as well as increased mitochondrial protein abundance and the activity of the key enzymes involved in the Krebs cycle in C2C12 myotubes [117]. GSK-3β KO mice showed augmented unloading-induced decreases in PGC-1α gene expression and its downstream targets in gastrocnemius muscle compared to wild-type animals [117]. Gastrocnemius muscles of GSK-3β KO mice were also protected against the unloading-induced decrements in protein and mRNA levels of respiratory chain enzymes and nuclear respiratory factor-1 (NRF-1) protein content [118].

In 2020, it was shown that constitutively active GSK-3α Ser 21A/GSK-3β Ser 9A knock-in mice had lower levels of PGC-1α downstream targets than WT animals, and PGC-1α mRNA expression did not decrease in soleus muscles of knock-in mice in response to 14 days HS compared to WT animals [71]. Surprisingly, time-course changes of PGC-1α downstream targets expression restoration during a period of recovery from unloading was similar between WT and knock-in animals, except for NRF-1, NRF-2α and TFAM that were more potently induced during early reloading in the knock-in animals. Earlier the same research group showed that muscle-specific knockout of GSK-3β enhances PGC1α, TFAM, NRF-1, and NRF-2α expression during early reloading [117]. This discrepancy could be caused by GSK-3 Ser phosphorylation-independent effects of reloading or by the differences in the signaling networks involved in the regulation of relatively slow soleus muscle and fast-type gastrocnemius muscles.

**Figure 4:**
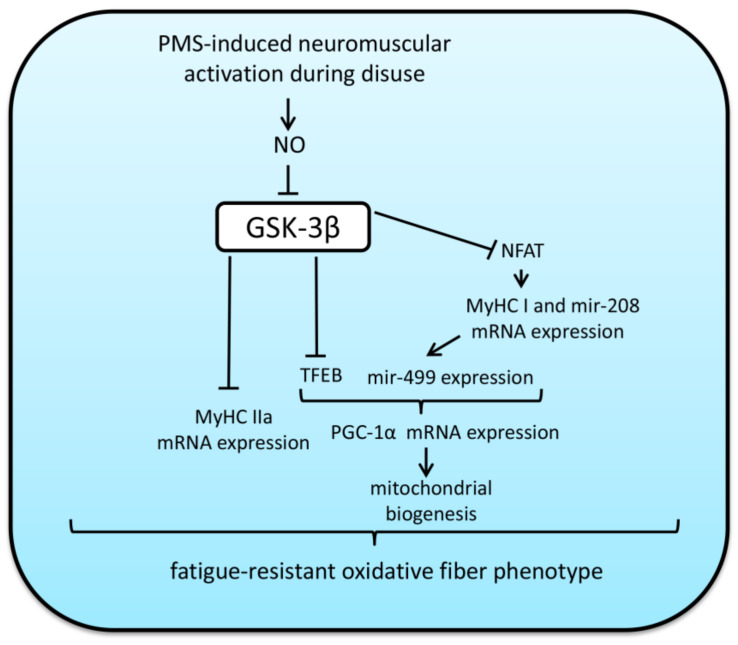
Regulation of myosin phenotype and oxidative capacity by GSK-3β in unloaded rat soleus muscle. Arrows indicate stimulatory signals, whereas T bars represent inhibitory signals. Plantar mechanical stimulation (PMS) leads to GSK-3β inactivation via NO-dependent pathway. GSK-3β blocks transcriptional activities of NFAT and TFEB transcription factors that can lead to downregulation of both PGC-1α and slow MyHC I and subsequent decrease in mitochondrial biogenesis. Thus, NO-dependent inactivation of GSK-3β facilitates fatigue-resistant oxidative phenotype of skeletal muscle fibers. NO—nitric oxide, MyHC—myosin heavy chain, PKG—protein kinase G, NFAT– nuclear factor of activated T-cells, mir-208—micro-RNA 208, TFEB—transcription factor EB, PGC1α—peroxisome proliferator-activated receptor gamma coactivator 1-alpha, MyHC—myosin heavy chain.

Recently, we have observed that L-arginine administration during 7-day rat HS as well as 3-day PMS prevented a decrease in both GSK-3β Ser9 phosphorylation and PGC-1α mRNA content [50,114]. The mechanisms of this effect remain unclear. However, it is well known that MyHC I mRNA transcription can also affect mitochondrial biogenesis. In the process of MyHC I mRNA transcription also occurs the expression of microRNA 208b, encoded in one of the introns of this gene. MicroRNA 208b is able to induce the expression of the gene myh7b, which is an ancient gene of slow-tonic myosin isoform. Myh7b protein is largely absent in the muscle fiber of postural muscles in humans and rats and its mRNA plays only regulatory role. mRNA of the myh7b gene encodes regulatory micro-RNA 499 [119]. Micro-RNA 499 and micro-RNA 208b bind to the 3’-untranslated region of transcriptional repressors of slow-type genes, such as SOX6, PuRA, PuRB, THRAP-1, and SP3 blocking their expression [79,83,120] and leading to the activation of transcription of genes to which these repressors bind. Transcription of myh7b mRNA (as well as MyHC I) is significantly reduced in rat soleus muscles after 24 h of HS [121] and remains decreased after 3 and 7-day HS. SOX6 mRNA elevates after the 3rd day of HS and remains increased after 7-day unloading [66,122]. It was shown that overexpression of microRNA-499 in C2C12 myotubes leads to an increased expression of the slow isoform of troponin (TNNI1), myoglobin, and PGC-1α, thus this microRNA plays an important role in the realization of the oxidative phenotype of muscle fiber (Xu, Chen et al. 2018). In our laboratory, it was found out that both PMS and L-arginine administration can prevent myh7b/mir-499 downregulation, SOX6 mRNA accumulation as well as PGC-1α decline in rat soleus during 7-day rat HS [114,121]. As both interventions are able to downregulate GSK-3β activity, it is possible that GSK-3β can control PGC-1α expression by affecting MyHC I/mir-499/SOX6 signaling pathway. However, in 2020, it was shown that GSK-3β inactivation in C2C12 myotubes can lead to the activation of transcription factor EB (TFEB), which, in turn, can activate PGC-1α mRNA transcription. Knockdown of TFEB completely prevented increases in PGC-1α gene expression induced by GSK-3β inactivation [123]. GSK-3β also indirectly regulates myocyte enhancer factor-2 (MEF-2) transcription activity in skeletal and cardiac muscle by a cross-talk with p38 MAP kinase [124], thus, this mechanism could also contribute to the effect of GSK-3β on both MyHC isoforms and PGC-1α expression. Other potential mechanisms of GSK-3β-dependent PGC-1α regulation include GSK-3-dependent inhibition of AMPK [33] (a well-known activator of PGC-1α) or phosphorylation of PGC-1α labeling it for proteasomal degradation [125]. However, currently there are no clear evidences indicating that these mechanisms could significantly contribute to the unloading-induced PGC-1α downregulation. Schematic diagram illustrating the role of GSK-3β in the regulation of MyHC mRNA expression and mitochondrial biogenesis via PMS-induced NO production in rat postural muscle is presented in Figure 4.

Thus, GSK-3β can serve as a key regulator promoting the formation of fast-type glycolytic fiber phenotype by co-regulating a decrease in transcription of both slow-type myosin and mitochondrial genes. Hence, it is not surprising that inhibition of GSK-3β can lead to changes in muscle performance. Indeed, in a recent report by Whitley and colleagues (2020) it was shown that inhibition of GSK-3 with low-dose LiCl (10 mg/kg per day for 6 weeks) enhanced muscle fatigue resistance and specific force production in murine soleus muscle, which was accompanied by increases in PGC-1α and MyHC I protein expression [126]. However, a potential of GSK-3 inhibition as a countermeasure against unloading-induced changes in the contractile properties of postural muscles is yet to be determined.

In humans, mechanical unloading (10-day bedrest) can induce a significant decline in the content of mitochondrial respiratory chain enzymes [99] as well as PGC1-1α expression in vastus lateralis muscle [101]. Recent proteomics data revealed that downregulation of the mitochondrial respiratory chain enzymes continues up to 21-day bedrest [127]. Slow-to-fast fiber-type transformation occurs after both 84 days [128] and 35 days of bedrest [129]. However, the lack of phospho-proteomics data on disused human skeletal leads to a lack of knowledge about GSK-3 activity at different stages of bedrest, so we can only assume that, as in animal models, under disuse conditions GSK-3 in human skeletal muscles may be activated at some time-points. Thus, based on this assumption, we can suggest that under unloading/disuse the downregulation of respiratory chain enzymes and slow-to-fast fiber-type transformation in human skeletal muscle could be, at least partially, mediated by GSK-3.

Unfortunately, most of the data concerning the roles of GSK-3 in various aspects of muscle disuse are obtained in rodent models. The limitations and problems of translation the results from rodents to humans were discussed and reviewed elsewhere [130,131,132]. However, in general, the key disuse-induced functional changes of skeletal muscles as well as the principal intracellular signaling alterations related to disuse are similar between rodents and humans, although the speed of these changes may vary.

While our review is focused on the roles of GSK-3 in disuse-induced muscle transformations, it is worth noting that GSK-3 can be involved in the development of multi-systemic pathological conditions. In particular, in has been demonstrated that an increased activity of GSK-3 occurs during myotonic dystrophy type 1, an autosomal dominant disorder affecting skeletal muscles as well as eyes, heart, endocrine, and nervous systems [133]. It was shown that small molecular GSK-3 inhibitors can reduce gastrocnemius muscle atrophy and grip strength decline in a mouse model of myotonic dystrophy type 1 by targeting GSK-3/cyclin D 3/cyclin D-dependent kinase 4/CUG-binding protein 1 pathway [133]. It was also demonstrated that GSK-3 inhibition is able to normalize GSK-3/CUG-binding protein 1 pathway in human myoblasts from patients with myotonic dystrophy type I and congenital myotonic dystrophy type I [134]. GSK-3 inhibitors were also shown to have a potential for preventing glucocorticoid-induced muscle degeneration [135] as well as skeletal muscle wasting related to pulmonary inflammation [136].

## 6. Conclusions and Perspectives

Accrued evidences indicate that GSK-3β activity plays a crucial role in skeletal muscle metabolism both in health and under atrophic conditions. Novel findings linking the activity of GSK-3β to the regulation of such vital processes as protein synthesis/ribosome biogenesis, breakdown of myofibrillar proteins, mitochondrial biogenesis and expression of slow MyHC have steadily emerged during the last 5–10 years in the field of skeletal muscle physiology. While many studies associate unloading/reloading-induced changes in GSK-3β with signaling pathways implicated in the regulation of myosin phenotype, protein turnover and oxidative capacity, still remain several unanswered questions. For example, what specific molecular mechanisms, apart from Ser9, could be implicated in the regulation of GSK-3β activity in skeletal muscle during disuse and subsequent reloading (the role of S-nitrosylation, subcellular localization, etc.). While recent studies showed that GSK-3β activity under disuse conditions depends upon NO-signaling, it is pertinent to investigate the role of NO-dependent regulation of GSK-3β in skeletal muscle during recovery from a period of mechanical unloading. Moreover, given that preliminary studies suggest that GSK-3β might be involved in the regulation of ribosome biogenesis during 7-day mechanical unloading, it is important to continue research into possible implication of GSK-3β in the regulation of translational capacity both under disuse conditions and a period of recovery.

Currently, there is a paucity of studies that use various available GSK-3β inhibitors during periods of skeletal muscle inactivity in order to elucidate possible GSK-3β-related mechanisms regulating different aspects of intracellular signaling in muscle fibers. Hence, additional research is needed to test the effect of both lesser-used, but more selective non-ATP-competitive and substrate-competitive GSK-3 inhibitors as well as LiCl, a widely used FDA-approved magnesium-competitive GSK-3 inhibitor (although less selective) in skeletal muscle under disuse/unloading conditions. In spite of the fact that pharmacological modulation of GSK-3β by LiCl is widely used in humans for the treatment of psychiatric disorders, there is a lack of studies on possible therapeutic effects of GSK-3β inhibition on human skeletal muscles under disuse conditions.

Overall, due to its vital functions in muscle fibers, GSK-3β may represent a perspective therapeutic target in the treatment of skeletal muscle wasting induced by prolonged disuse, aging, and a number of chronic diseases.

## Figures and Tables

**Table 1:** The effects of unloading and reloading on GSK-3β (Ser9) phosphorylation in skeletal muscles.

	Unloading Conditions				
Species	Model	Muscle	Time-Point	Changes in GSK-3β	References
mouse	HU	soleus, plantaris	14 days	p-GSK-3β (Ser9) ─	[56]
rat	IM	soleus	10 days	p-GSK-3β (Ser9) ─	[59]
rat	HU	soleus	1, 3 days	p-GSK-3β (Ser9) ↓	[50]
rat	HU	soleus	3, 7 days	p-GSK-3β (Ser9) ↓	[51,52]
rat	HU	soleus	14 days	p-GSK-3β (Ser9) ↓	[52,54,55]
rat	HU	tibialis anterior	14 days	p-GSK-3β (Ser9) ─	[54]
rat	HU	soleus	28 days	p-GSK-3β (Ser9) ↓	[52]
rat	HU	soleus	38 days	p-GSK-3β (Ser9) ↓	[60]
rat	HU	gastrocnemius	38 days	p-GSK-3β (Ser9) ─	[60]
human	IM	quadriceps femoris	2 days	p-GSK-3β (Ser9) ↓	[69]
human	IM	quadriceps femoris	14 days	p-GSK-3β (Ser9) ─	[70]
Reloading conditions:					
Species	Model	Muscle	Time-point	Changes in GSK-3β	References
mouse	HU	soleus	3 and 5 days	p-GSK-3β (Ser9) ↑	[56]
mouse	HU	soleus	7 and 14 days	p-GSK-3β (Ser9) ─	[56]
mouse	HU	plantaris	3, 5, 7, 14 days	p-GSK-3β (Ser9) ─	[56]
rat	IM	soleus	6 and 15 days	p-GSK-3β (Ser9) ↑	[59]
rat	HU	soleus	3 days	p-GSK-3β (Ser9) ↑	[54,57,58]
rat	HU	soleus	7 and 14 days	p-GSK-3β (Ser9) ─	[54,57]
rat	HU	tibialis anterior	3, 7,14 days	p-GSK-3β (Ser9) ↑	[54]
rat	HU	soleus	14 days	p-GSK-3β (Ser9) ─	[55]
rat	HU	soleus	7 and 14 days	p-GSK-3β (Ser9) ─	[57]

↑ indicates a significant increase compared to control values; ↓ indicates a significant increase compared to control values; ─ indicates no change compared to control values. HU—hindlimb unloading, IM—limb immobilization, DI—dry immersion.

## Abbreviations

4E-BP1	eukaryotic initiation factor 4E binding protein
AKT	protein kinase B
AMPK	AMP-activated protein kinase
CaN	calcineurin
cGMP	cyclic guanosine monophosphate
c-Myc	c-myelocytomatosis oncogene (transcription factor)
CSA	cross-sectional area
eIF2B	eukaryotic initiation factor 2B
FoxO	forkhead box O protein
GC	guanylate cyclase
GS1	glycogen synthase-1
GSK-3β	glycogen synthase kinase-3β
GSK-3β KO	mice lacking muscle GSK-3β
HS	hindlimb suspension
HU	hindlimb unloading
IGF-1	insulin-like growth factor 1
ILK	integrin-linked kinase
IR	insulin/insulin-like growth factor 1 receptor
IRS-1	insulin receptor substrate 1
LC3	microtubule-associated proteins 1A/1B light chain 3B
LiCl	lithium chloride
L-NAME	N(gamma)-nitro-L-arginine methyl ester
MAFbx	muscle atrophy F-box protein/atrogin-1
MAPK	mitogen-activated protein kinase
MCIP 1.4	modulatory calcineurin-interaction protein 1.4
MEF-2	myocyte enhancer factor-2
mir-208	micro-RNA 208
mTORC1	mammalian/mechanistic target of rapamycin complex 1
MuRF1	muscle RING finger protein
MyHC	myosin heavy chain
NES	nuclear export signal
NFAT	nuclear factor of activated T-cells
NO	nitric oxide
NRF	nuclear respiratory factor
p70S6K	ribosomal protein S6 kinase p70
PGC-1α	peroxisome proliferator-activated receptor gamma coactivator 1-alpha
PI3K	phosphatidylinositol 3-kinase
PKA	protein kinase A
PKB	protein kinase B (AKT)
PKC	protein kinase C
PKD1	protein kinase D1
PKG	protein kinase G
PMS	plantar mechanical stimulation
PP1	protein phosphatase 1
PP2A	protein phosphatase 2A
PuRA	purine Rich Element Binding Protein A
PuRB	purine Rich Element Binding Protein B
rRNA	ribosomal RNA
SOX-6	transcription factor
SP3	transcription factor
TDZD-8	thiadiazolidinone-8
TFAM	Mitochondrial transcription factor A
TFEB	transcription factor EB
THRAP	mediator of RNA polymerase II transcription subunit 13
TNNI1	slow isoform of troponin
TSC2	tuberous sclerosis complex 2
UPP	ubiquitin–proteasome pathway
WT	wild-type mice
